# Zoonotic *Bartonella* Species in Cardiac Valves of Healthy Coyotes, California, USA

**DOI:** 10.3201/eid2012.140578

**Published:** 2014-12

**Authors:** Spencer P. Kehoe, Bruno B. Chomel, Matthew J. Stuckey, Rickie W. Kasten, Nandhakumar Balakrishnan, Benjamin N. Sacks, Edward B. Breitschwerdt

**Affiliations:** University of California, Davis, Davis, California, USA (S.P. Kehoe, B.B. Chomel, M.J. Stuckey, R.W. Kasten, B.N.Sacks);; North Carolina State University, Raleigh, North Carolina, USA (N. Balakrishnan, E.B. Breitschwerdt)

**Keywords:** Bartonella, Canis latrans, coyotes, cardiac valves, endocarditis, zoonoses, bacteria, vector-borne infections

Bartonellae are vector-borne gram-negative, aerobic, intracellular bacteria with a tropism for erythrocytes and endothelial cells (*1*). These bacteria, many of which are zoonotic, infect a wide range of domestic and wild animal species, causing a spectrum of disease manifestations and pathologies (*2*). Bartonellae, especially *Bartonella vinsonii* subsp. *berkhoffii* (*B. v. berkhoffii*), cause valvular endocarditis, especially of the aortic valve in mammals, including humans, dogs, cats, and cattle (*1*,*3*). Fleas and possibly ticks can vector *B. v. berkhoffii* (*4*). *Bartonella* species, typically observed in 5- to 7-year-old mid-sized to large dogs, account for ≈28% of endocarditis in dogs (*3*,*5*). Bartonellae, including *B. v. berkhoffii*, account for ≈3% of human endocarditis cases (*1**,**6*). In dogs and humans, these bacteria appear to have a specific tropism for aortic and mitral valves (*1*). Similar to lesions that develop with *Coxiella burnetii* endocarditis (*7*), valvular vegetative lesions can result from chronic *Bartonella* infection.

In California, coyotes (*Canis latrans*) are a major reservoir for *B. v. berkhoffii* (*8*). Natural *Bartonella* reservoir hosts are often asymptomatic, but to our knowledge, the possible role of *Bartonella*-induced endocarditis in coyotes has never been investigated. We hypothesized that *B. v. berkhoffii* or other *Bartonella* species could cause endocarditis in coyotes. We also hypothesized that bartonellae might preferentially localize to the aortic and/or mitral valves before vegetative lesions develop. Hence, coyotes served as a naturally occurring epidemiologic and physiologic sentinel model for studying infection kinetics and pathology induced by this bacterium in a reservoir host (coyotes).

## The Study

During the early 2000s, a total of 70 coyotes trapped in 9 northern California counties as part of a depredation control program were assessed for zoonotic heartworm (*Dirofilaria immitis*) disease (*9*). Coyote hearts and spleens were collected at that time and stored at –70°C in a manner to avoid DNA carryover during handling, storage, and processing. In 2012 and 2013, the hearts were dissected for macroscopic evidence of aortic and mitral valve vegetative endocarditis lesions. A board-certified veterinary pathologist examined possible valvular lesions or thickened regions; however, because the samples had been frozen, microscopic histopathologic examination was not conducted. We extracted DNA from aortic and mitral valves and spleens using DNeasy Blood and Tissue Kits (QIAGEN, Hilden, Germany). *B. v. berkhoffii*–spiked rabbit blood was the DNA extraction positive control. We tested extracted samples by PCR for *Bartonella* DNA targeting the citrate synthase gene (*glt*A) (*10*). PCR of spleen tissue was a substitute for blood culture detection of bacteremia. *B. henselae* DNA and distilled water were PCR-positive and -negative controls, respectively. Partial gene sequencing was performed on PCR-positive tissues. Nineteen aortic valve, mitral valve, and splenic DNA samples from 14 coyotes (*B. v. berkhoffii* PCR-positive animals by *gltA* PCR and sequencing) were genotyped by using primers targeting 16–23S intergenic transcribed spacer (ITS) region, as previously described (*11*) with minor modifications in annealing temperature (68°C for 15 s) and extension (72°C for 18 s). We conducted statistical analysis for differences in tissue tropism using Epi Info version 6 (Centers for Disease Control and Prevention, Atlanta, GA, USA).

Of the 70 coyotes collected from 9 counties (Figure), 45 (64%) were male. Coyotes’ ages ranged from <1 year (57 [81%]) to >5 years (3 [4%]). Nine (20%) male and 6 (24%) female coyotes were PCR positive for *Bartonella* species. Fourteen (93%) of the 15 *Bartonella*-positive coyotes were <3 years old, of which 13 (87%) were <1 year old. Prevalence by county ranged from 0% to 33% (Figure).

**Figure:**
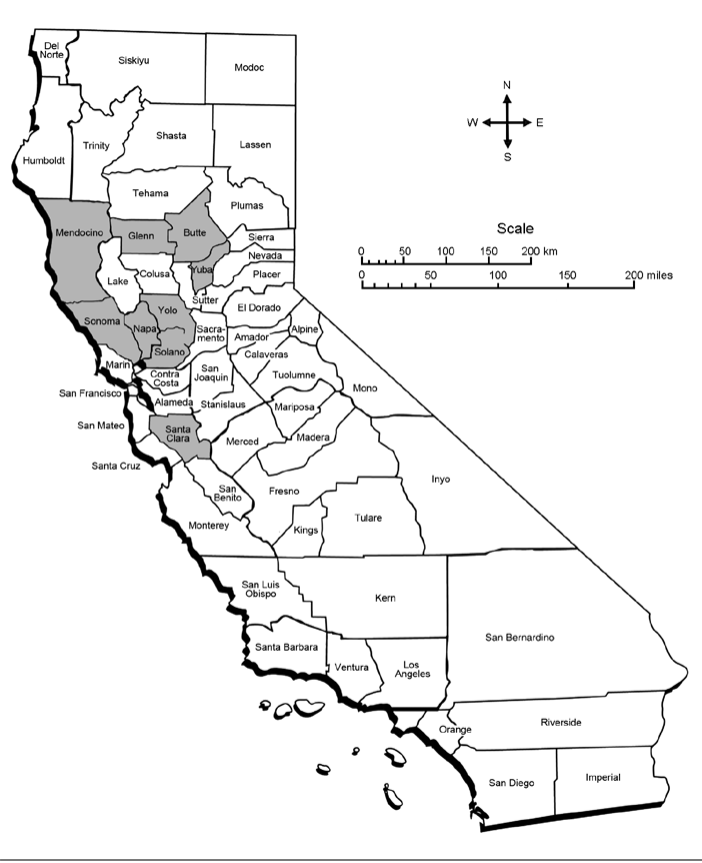
Molecular prevalence of *Bartonella* species in 70 coyotes from 9 counties, California, USA. Shaded areas are counties where coyotes were trapped during the early 2000s. *Bartonella*-positive coyotes were identified from the 9 counties as follows: Yuba, 6 (33%) of 18 trapped coyotes; Santa Clara, 3/22 (14%); Mendocino, 2/11 (18%); Napa, 2/6 (33%); Sonoma, 1/5 (20%); Glenn, 1/4 (25%); Yolo, 0/1; Butte, 0/1; Solano, 0/2.

We found no gross vegetative aortic or mitral valvular endocarditis lesions. Fifteen (21%) coyotes tested positive by PCR for *Bartonella* *gltA* gene. Overall, 8 aortic valves, 5 mitral valves, and 4 spleens were PCR positive. Aortic and mitral cardiac valves of 1 coyote (no. 93) tested positive by PCR for *B. v. berkhoffii,* and the aortic valve and spleen of another coyote (no. 110) were PCR positive (Table). Although a higher percentage of positive cardiac valves were aortic (53%) than mitral (33%), the difference was not significant. However, when we compared the number of *Bartonella*-infected cardiac valves (11 valves) with *Bartonella*-infected spleens (3 spleens), we found that *Bartonella* DNA was amplified 4.16 times (95% CI 1.02–24.12) more often from cardiac valves than from spleens.

**Table:** Coyotes (*Canis latrans*) positive for *Bartonella* species by PCR, California, USA

Coyote no.	Sex/estimated age, y	Weight, kg	County	*Bartonella* PCR-positive tissue	*Bartonella* species by DNA sequencing
91	F/1	11.7	Yuba	Aortic valve	*B. vinsonii* subsp. *berkhoffii* type III
92	M/1	13	Yuba	Aortic valve	*B. vinsonii* subsp. *berkhoffii* type I
93	M/1	10.5	Mendocino	Aortic valve	*B. vinsonii* subsp. *berkhoffii* type I
				Mitral valve	*B. vinsonii* subsp. berkhoffii*
99	M/<1	10.6	Yuba	Spleen	*B. rochalimae*
101	M/1	12.3	Yuba	Mitral valve	*B. vinsonii* subsp. berkhoffii*
102	M/<1	10	Glenn	Spleen	*B. vinsonii* subsp. *berkhoffii* type II
106	F/1	12	Yuba	Aortic valve	*B. vinsonii* subsp. berkhoffii*
110	M/<1	11	Yuba	Aortic valve, spleen	*B. vinsonii* subsp. *berkhoffii* type II
121	F/<1	8.6	Santa Clara	Aortic valve	*B. vinsonii* subsp. berkhoffii*
124	F/9	10.4	Santa Clara	Aortic valve	*B. vinsonii* subsp. berkhoffii*
137	M/<1	10.7	Mendocino	Mitral valve	*B. henselae*
146	M/3	16.6	Sonoma	Aortic valve	*B. vinsonii* subsp. *berkhoffii* type I
152	M/<1	9.7	Napa	Spleen	*B. vinsonii* subsp. *berkhoffii* type II
156	F/<1	8.1	Santa Clara	Mitral valve	*B. vinsonii* subsp. berkhoffii*
164	F/<1	10.1	Napa	Mitral valve	*B. vinsonii* subsp. *berkhoffii* type I
*Type not amplified.					

Partial DNA sequencing showed that aortic valves from 8 (53%) of 15 coyotes were *B. v. berkhoffii* positive, compared with mitral valves from 4 (27%) and spleens from 3 (20%) coyotes. *B. rochalimae* was amplified from the spleen of coyote no. 99, and *B. henselae* DNA was amplified from the mitral valve of coyote no. 137 (Table). Of 14 coyotes tested for *B. v. berkhoffii* genotypes by 16–23S ITS PCR, 8 were positive, whereas *Bartonella* DNA was not amplified from the remaining 6 tissue DNA samples by using ITS primers. By sequence analyses, 4 coyotes were infected with *B. v. berkhoffii* genotype I, 3 with genotype II, and 1 with genotype III.

## Conclusions

Our study documents the presence of 3 zoonotic *Bartonella* species in heart valves and/or spleen of free-ranging coyotes from northern California. Despite the absence of gross vegetative endocardial lesions, *Bartonella* DNA was amplified and sequenced from >20% of the coyotes, mainly from cardiac valves; only 4 (6%) coyotes had PCR-positive spleens, compared with 12 (17%) coyotes with PCR-positive cardiac valves. We hypothesize that *Bartonella* in the spleen indicated early or ongoing bacteremia, whereas bartonellae in the heart valves, in their absence in the spleen, indicated valvular bacterial localization, possibly facilitating persistent infection that could evolve through time to endocarditis. This evolution has been observed for *C. burnetii* infection in humans (*12*), for which the mean reported interval from illness onset to endocarditis diagnosis is 12–24 months (*7*). *Bartonella* endocarditis is usually seen in middle-aged dogs (mean age 6.3 years ± 2.8) (*3*,*5*) and in adult humans (mean age 54 years ± 16) (*6*). Because 93% of the PCR-positive coyotes were <3 years old, they were very likely too young for vegetative endocarditis to have developed.

Nevertheless, the fact that ≈20% of the cardiac valve tissues were PCR positive for *Bartonella* perhaps indicates that the bacteria had localized to the valves of infected coyotes. *B. v. berkhoffii* can induce vasoproliferative lesions in animals (*13*); thus, cases of *Bartonella* endocarditis might represent only a small fraction of infected animals that have chronic cardiac valvular localization. All 3 *B. v. berkhoffii* genotypes identified in these coyotes have been previously involved in humans or dogs with endocarditis (*14*,*15*). To our knowledge, *B. henselae* and *B. v. berkhoffii* genotype III have not been previously identified in coyotes; thus, these mammals can be added to the list of susceptible species. Coyotes might be a natural reservoir for *B. v. berkhoffii* genotype III, which so far has been mainly described in California gray foxes (*14*).

In conclusion, *Bartonella* infection of a natural reservoir appears to lead to cardiac valve tropism. This tropism could result in development of endocarditis, a severe and often lethal complication of *Bartonella* infection.
